# A comparison of machine learning classifiers for dementia with Lewy bodies using miRNA expression data

**DOI:** 10.1186/s12920-019-0607-3

**Published:** 2019-10-30

**Authors:** Daichi Shigemizu, Shintaro Akiyama, Yuya Asanomi, Keith A. Boroevich, Alok Sharma, Tatsuhiko Tsunoda, Takashi Sakurai, Kouichi Ozaki, Takahiro Ochiya, Shumpei Niida

**Affiliations:** 1 Laboratory Chief, Division of Genomic Medicine, Medical Genome Center, National Center for Geriatrics and Gerontology, 7-430 Morioka-cho, Obu, Aichi 474-8511 Japan; 2 Department of Medical Science Mathematics, Medical Research Institute, Tokyo Medical and Dental University (TMDU), Tokyo, 113-8510 Japan; 3 RIKEN Center for Integrative Medical Sciences, Yokohama, Kanagawa 230-0045 Japan; 4 CREST, JST, Tokyo, 113-8510 Japan; 5 School of Engineering & Physics, University of the South Pacific, Suva, Fiji; 6 Institute for Integrated and Intelligent Systems, Griffith University, QLD, Brisbane, 4111 Australia; 7 The Center for Comprehensive Care and Research on Memory Disorders, National Center for Geriatrics and Gerontology, Obu, Aichi 474-8511 Japan; 8 Department of Cognitive and Behavioral Science, Nagoya University Graduate School of Medicine, Nagoya, Aichi 466-8550 Japan; 9 Division of Molecular and Cellular Medicine, Fundamental Innovative Oncology Core Center, National Cancer Center Research Institute, Tokyo, 104-0045 Japan; 10 Institute of Medical Science, Tokyo Medical University, Tokyo, 160-8402 Japan

**Keywords:** Dementia with Lewy bodies, Risk prediction model, microRNAs, Single nucleotide polymorphism

## Abstract

**Background:**

Dementia with Lewy bodies (DLB) is the second most common subtype of neurodegenerative dementia in humans following Alzheimer’s disease (AD). Present clinical diagnosis of DLB has high specificity and low sensitivity and finding potential biomarkers of prodromal DLB is still challenging. MicroRNAs (miRNAs) have recently received a lot of attention as a source of novel biomarkers.

**Methods:**

In this study, using serum miRNA expression of 478 Japanese individuals, we investigated potential miRNA biomarkers and constructed an optimal risk prediction model based on several machine learning methods: penalized regression, random forest, support vector machine, and gradient boosting decision tree.

**Results:**

The final risk prediction model, constructed via a gradient boosting decision tree using 180 miRNAs and two clinical features, achieved an accuracy of 0.829 on an independent test set. We further predicted candidate target genes from the miRNAs. Gene set enrichment analysis of the miRNA target genes revealed 6 functional genes included in the DHA signaling pathway associated with DLB pathology. Two of them were further supported by gene-based association studies using a large number of single nucleotide polymorphism markers (BCL2L1: *P* = 0.012, PIK3R2: *P* = 0.021).

**Conclusions:**

Our proposed prediction model provides an effective tool for DLB classification. Also, a gene-based association test of rare variants revealed that BCL2L1 and PIK3R2 were statistically significantly associated with DLB.

## Background

Dementia with Lewy bodies (DLB) is the second most common subtype of neurodegenerative dementia in humans following Alzheimer’s disease (AD) [1] and accounts for around 4.6% of all dementia cases [2]. The main pathological lesions in DLB are Lewy bodies and neurites, containing abnormal α-synuclein (α Syn) [3]. The characteristic features of DLB are different to those in AD, with less marked memory impairment and more severe impairments of visuospatial, attentional and frontal-executive functions [3].

Present clinical diagnosis of DLB has high specificity and low sensitivity [4], and finding DLB patients in the prodromal phase is still challenging. An accurate diagnosis of DLB at the prodromal stage would be an important advance in the pharmacological management, as cholinesterase inhibitors (ChEIs) have good responsiveness for patients with Lewy body dementia (LBD) including DLB and Parkinson’s disease dementia (PDD), although a careful monitoring of treatment compliance and side effects is required [5]. Therefore, as potential biomarkers for prodromal DLB are required in clinical implication, our findings might enable DLB to be one of the most treatable neurodegenerative disorders.

MicroRNAs (miRNAs) are small non-coding RNAs, which play key roles in many biological or pathological processes by regulating the expression of their target transcripts. Previous studies have reported that alterations in miRNA expression have be associated with several neurodegenerative diseases [6–8]. We also reported potential biomarkers for earlier diagnosis and therapeutic intervention through comprehensive miRNA expression analyses and constructed a risk prediction model using the biomarkers based on supervised principal component analysis (PCA) logistic regression, a machine learning (ML) method [9].

Current studies for disease prediction models implemented several ML methods. For example, Lebedev et al. reported a random forest model that predicted MCI-to-AD conversion with high accuracy using morphometric measures from 3D brain MRI images and clinical information [10]. Wei et al. developed a promising support vector machine method that detected persons with diabetes and pre-diabetes using comprehensive clinical information [11]. We have also reported several efficient risk prediction models for not only type II diabetes based on a penalized regression method (LASSO) incorporating clinical information and genetic data [12], but also postoperative overall survival and disease-free survival in patients with breast cancer based on a Cox proportional hazard model [13]. However, there is, as of yet, no clear consensus of which ML method is most appropriate for application to disease prediction models.

Here, we applied several ML methods to comprehensive miRNA expression data of serum samples, composed of DLB patients and individuals with cognitive normal function (referred to as normal controls: NC) and investigated an optimal risk prediction model from among these ML methods. We used 10-fold cross-validation on a training set consisting of half of the Japanese individuals, separated from a test set. We constructed risk prediction models using four ML methods, penalized regression [14–16], random forest (RF) [17], support vector machine (SVM) [18], and gradient boosted decision tree (GBDT) [19], and evaluated the predictive performance of the ML models on an independent test set. This final model based on GBDT showed better accuracy than the model based on supervised PCA logistic regression constructed in our previous studies [9].

## Methods

### Clinical samples

All of 457 serum subjects and their clinical data were obtained from the National Center for Geriatrics and Gerontology (NCGG) Biobank. The total set of subjects was composed of 169 DLB patients and 288 normal control (NC) subjects with normal cognitive function [9]. The DLB subjects were diagnosed on basis of the criteria of the fourth report of the DLB Consortium [20]. The NC subjects were confirmed with a Mini-Mental State Examination (MMSE) score ≥ 27. The APOE ε_4_ genotype and MMSE score of all subjects were available. These miRNA expression data are publicly available through the Gene Expression Omnibus (GEO) database at the National Center for Biotechnology Information (GSE120584, http://www.ncbi.nlm.nih.gov/projects/geo/).

Sixty-nine DLB cases and 2008 NCs used in the genetic association studies were also selected from the subjects enrolled in the NCGG Biobank. All subjects were ≥ 60 years in age and were genotyped using Japonica arrays [21]. We excluded all SNPs with a genotype call rate < 0.99, a Hardy-Weinberg equilibrium *p*-value < 1.0 × 10^−3^ in NCs or a minor allele frequency (MAF) < 0.01.

### Target gene annotation using miRNAs

The target genes of miRNAs were determined using the microRNA Target Prediction and Functional Study Database (miRDB version 5.0, [22], where MirTarget V3 predicted the miRNA-target genes with a prediction score in the range between 0 and 100. In this study, target genes with a score of > 90 were used in further gene-based association studies.

### Parameter selection in several machine learning methods

Top-ranked miRNAs were detected using a logistic regression method after adjustment for age, sex and APOE ε_4_ genotype on the training set. All ML model optimizations were performed against each pre-selected top-ranked p miRNA using 10-fold cross-validation on the training set. The hyper-parameter optimizations were implemented in the *scikit-learn* library (version 0.19.1) in Python. Precision, recall, F-measure, and accuracy were used to evaluate the four ML methods: penalized regression, RF, SVM, GBDT. The precision, recall, F-measure, and accuracy were calculated using the counts of true positives (TP), true negatives (TN), false positives (FP), and false negatives (FN) as follows:

$$ Precision=\frac{TP}{TP+ FP} $$

$$ Recall=\frac{TP}{TP+ FN} $$

$$ F- measure=\frac{2 Recall\bullet Precision}{Recall+ Precision} $$

$$ Accuracy=\frac{TP+ TN}{TP+ TN+ FP+ FN} $$

### Random forest (RF)

Using the best prediction model based on random forest method, we investigated the optimal combination of the following five hyper-parameters: *max*_*depth*_*, n*_*tree*_*, min*_*split*_, *min*_*sample*_, and *criterion.* The *max*_*depth*_ parameter is maximum number of levels in each decision tree (*max*_*depth*_ = 2, 3, 4, 5). The *n*_*tree*_ parameter is the number of decision trees (*n*_*tree*_ = 100, 200, 300, 400, 500, 600, 700, 800, 900, and 1000). The *min*_*split*_ parameter is minimum number of data points placed in a node before the node is split (*min*_*split*_ = 2, 3, 5, 10, 15, 20). The *min*_*sample*_ parameter is the minimum number of samples in a leaf (*min*_*sample*_ = 1, 3, 5). The *criterion* parameters, ‘*gini impurity*’ and ‘*information gain entropy*’, are used for splitting the data.

### Penalized regression

We implemented ridge regression [14], elastic net [15] and lasso methods [16], known as penalized regression methods. The phenotype of subject *i* = 1, ⋯, *n* was set as the dependent variables (case = 1, control = 0) and the expression *X*_*i*, *j*_ of each miRNA *j* = 1, ⋯, *m* for a subject *i*. Let *X*_*i*_ = (*X*_1_, ⋯, *X*_*p*_) be the values of pre-selected top-ranked *p* miRNA for a subject *i* and let *l*(*β*; *γ*_*i*_, *X*_*i*_) be the logistic log-likelihood:

$$ l\left(\beta; {\gamma}_i,{X}_i\right)-\lambda {P}_{\alpha}\left(\beta \right), $$

where ${P}_{\alpha}\left(\beta \right)=\left(1-\alpha \right)\frac{1}{2}{\beta}^2+\alpha \mid \beta \mid$, and *α* was set to 1 for lasso, 0 for ridge regression, and 0 to 0.9 at 0.1 intervals for elastic net, and *λ* are selected using 10-fold cross-validation. For the best prediction model based on penalized regression methods, we investigated optimal combinations of above two hyper-parameters: *α* and *λ*.

### Support vector machine (SVM)

To construct prediction models, we applied a support vector machine method with the radial basis function (RBF) kernel defined as:

$$ K\left(x,x^{\prime}\right)=\mathit{\exp}\left(-\gamma {\left\Vert x-x\prime \right\Vert}^2\right) $$

$$ \underset{\beta, \xi }{\min}\frac{1}{2}{\left\Vert \beta \right\Vert}^2+C\sum \limits_{i=1}^n{\xi}_i $$

We investigated the optimal combinations of the above two hyper-parameters: *gamma* (γ) and *cost* (*C*). The parameters *C* and *γ* affect model complexity and model smoothness, respectively. Increasing C and *γ* cause over-fitting, and range of those parameters we implemented were *C* = {2^−15^, 2^−14^, …, 2^14^, 2^15^} and *γ* = {2^−15^, 2^−14^, …, 2^14^, 2^15^}. The terms *β* and $\sum \limits_{i=1}^n{\xi}_i$ were then coefficients of classifier resulting from a separating hyperplane and the amount of misclassified data.

### Gradient boosting decision tree (GBDT)

Significantly different from the similar ensemble method, random forest, the tree-based models of GBDT were trained sequentially, and each base model was updated to correct the error produced by its previous tree models, called a learning rate. To optimize parameters in this method, we examined combinations of the following five hyper-parameters: *max*_*depth*_*, n*_*tree*_*, min*_*split*_, *min*_*sample*_, and *learning rate.* The first four parameters were the same as those used in random forest. The learning rate was optimized across {0.001, 0.01, 0.05, 0.1, 0.2}.

## Results

### Data collection of Japanese individuals

We split the 457 Japanese individuals (169 DLB cases, 288 controls) into a training set of 229 individuals (85 DLB cases, 144 controls) and a test set of 228 individuals (84 DLB cases, 144 controls). This separation was performed to result in a similar distribution in the age between the training and test sets (Table 1).

**Table 1:** Average age, sex and APOE ε_4_ genotype information in the training and test data

	Training data set				Test data set			
Phenotype	#Sample	Age	Sex (Male)	APOE ε_4_^a^	#Sample	Age	Sex (Male)	APOE ε_4_^a^
DLB	85	79.5	0.45	0.34	84	79.5	0.36	0.30
NC	144	71.7	0.49	0.22	144	71.8	0.56	0.15

^a^APOE ε_4_ shows the average of the number of APOE ε_4_ genotype

### Comparison of classifier performance

**Fig. 1:**
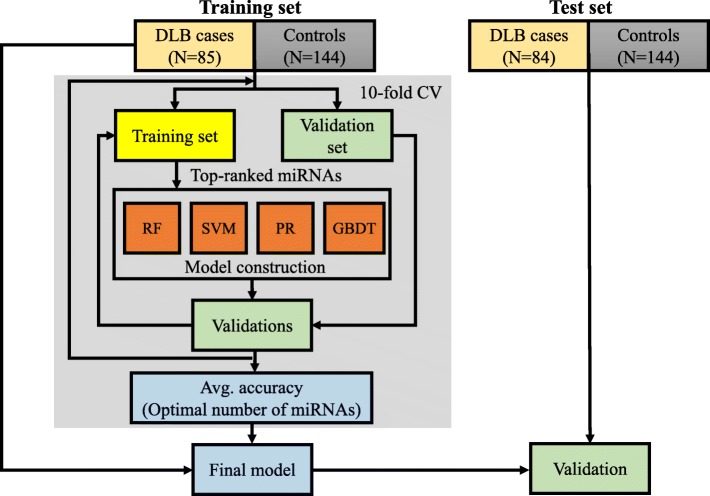
Outline of the risk prediction model construction and the validation

**Fig. 2:**
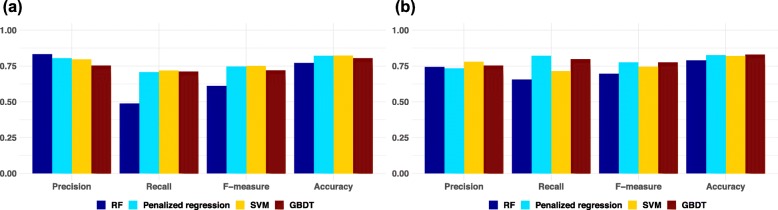
Precision, Recall, F-measure, and Accuracy values listed four ML methods. Performance of four ML methods on training (**a**) and test (**b**) data

All approaches were performed using a data set of the p most significant miRNAs in a stepwise manner (*p* ≤ 500). The most significant miRNAs (top-ranked miRNAs) were determined in nine-tenths of entire training set using a logistic regression method. Note that top-ranked miRNAs were determined for each cross-validation step. The adjusted model was constructed using the nine-tenths of the training set and was evaluated using the remaining one-tenth. Four ML methods, penalized regression (ridge regression, elastic net and least absolute shrinkage and selection operator: LASSO), RF, SVM, and GBDT, were used for model construction. Using 10-fold cross validation estimation, we determined the optimal number of miRNAs for the final model construction for each ML method (Fig. 1). Final models were constructed using the complete training set. The number of top-ranked miRNAs and the tuning hyper-parameters are shown in Table 2. The adjusted models constructed with the entire training set were then evaluated on a completely independent test set (Fig. 2). Among the four MLs, a final risk prediction model based on the GBDT method achieved the highest accuracy of 0.829 when pre-selecting the top-ranked 216 miRNAs and three clinical features. The other methods were 0.825 for penalized regression with 434 miRNAs, 0.820 for SVM with 27 miRNAs, and 0.789 for RF with 60 miRNAs (Table 2 and Fig. 2). The hyper-parameters used in the final risk prediction model with GBDT were then optimized: (*max*_*depth*_, *n*_*tree*_*, min*_*split*,_ *min*_*sample*_, *learning rate*) = (4, 200, 20, 5, 0.1) (see the Methods).

**Table 2:** Hyperparameter values in each final model

Method	#top-ranked miRNA	Hyperparameter	Value
Penalized regression	434	*α*	0.1
		*λ*	0.10882
RF	60	*max*_*depth*_	4
		*n*_*tree*_	100
		*min*_*split*_	10
		*min*_*sample*_	3
SVM	27	*C*	2.14355
		*γ*	0.001122
GBDT	216	*max*_*depth*_	4
		*n*_*tree*_	200
		*min*_*split*_	20
		*min*_*sample*_	5
		*learning rate*	0.1

We also constructed a GBDT risk prediction model using another feature selection algorithm, μHEM [23], publicly available at http://www.isical.ac.in/~bibl/results/mihem/mihem.html, and investigated whether this feature selection methodology can further improve the predictive ability of our model. The GBDT risk prediction model was performed using a data set of the top-ranked p miRNAs in a stepwise manner (*p* ≤ 500). This final risk prediction model using μHEM algorithm achieved an accuracy of 0.803 on an independent test set when pre-selecting the top-ranked 330 miRNAs and three clinical features. Although the final risk prediction model showed a lower accuracy than that using a logistic regression method (Additional file 1: Table S1), implementation of feature selection algorithms might contribute to further improvement of the GBDT risk prediction model.

**Fig. 3:**
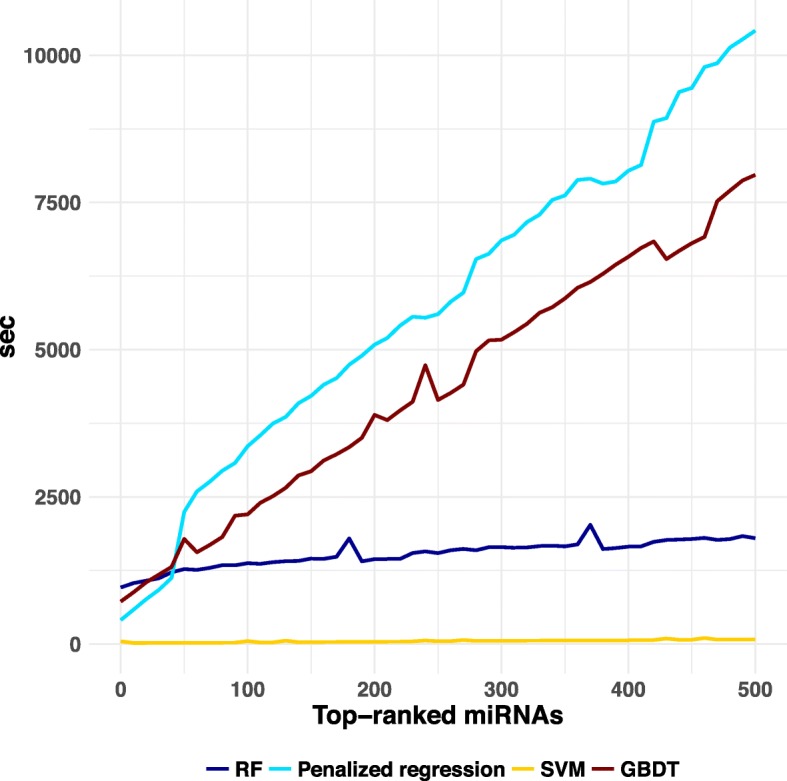
Runtimes of four ML methods

We also compared the run time of the four ML methods on a 7-core Intel Xeon 2.40GHz CPU with 256 GB of memory. While runtimes for SVM and RF were independent of the number of top-ranked miRNAs used, the runtimes for GBDT and penalized regression increased with the number of top-ranked miRNAs. SVM was the fastest ML method, which spent 1.5 min to construct the risk prediction model when using top-ranked 500 miRNAs. RF took 30 min, GBDT took 2.2 h, and penalized regression took 2.9 h (Fig. 3). This result shows that difficulties may arise when implementing GBDT and penalized regression with larger numbers of top-ranked miRNAs in the risk prediction model construction compared with SVM and RF.

### Effective features used in risk prediction model

The final GBDT risk prediction model was constructed by pre-selecting the 216 top-ranked miRNAs and three clinical features (‘age’, ‘APOE ε_4_ genotype’, and ‘sex’). Of the 219 features, 182 were used in the final risk prediction model construction as effective features with a feature importance > 0 (180 miRNAs and 2 clinical features: ‘age’ and ‘APOE ε_4_ genotype’) (Additional file 2: Table S2).

To examine the biological significance of our findings (180 miRNAs), we further predicted the miRNA functional target genes using miRDB [22]. The miRNAs were predicted to target 4119 genes (see the Methods), of which 423 genes were predicted by the 7 miRNAs (MIMAT0014984, MIMAT0027624, MIMAT0016852, MIMAT0023713, MIMAT0019849, MIMAT0022491, and MIMAT0007882) with a feature importance > 0.015 in the final GBDT risk prediction model (Additional file 2: Table S2). The rank of miRNAs’ feature importance in the final GBDT risk prediction model was also correlated with that of miRNAs chosen by the logistic regression method (Spearman’s *ρ* =0.21 and *p*-value = 0.006).

### Functional pathways using gene set enrichment analysis (GSEA)

**Fig. 4:**
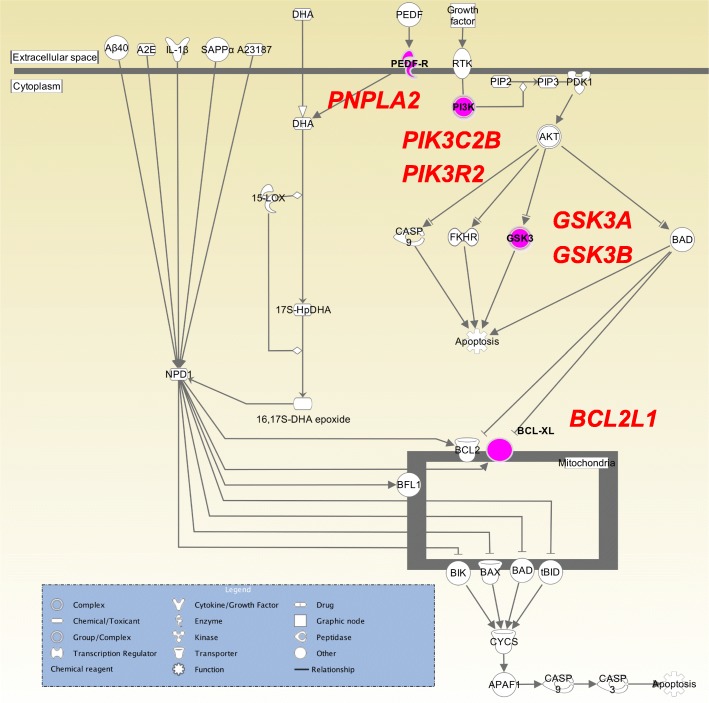
Docosahexaenoic acid (DHA) signaling pathway detected by GSEA. The DHA signalling pathway was generated through the use of IPA (QIAGEN Inc., https://www.qiagenbioinformatics.com/products/ingenuity-pathway-analysis)

In order to elucidate any enrichment of functional units or categories, we applied GSEA to the 423 target genes of the 7 miRNAs described above. GSEA was performed using Ingenuity Pathways analysis software (IPA; Ingenuity Systems). We identified six statistically significant canonical pathways: protein kinase A signaling (21 genes), ERK/MAPK signaling (14 genes), molecular mechanisms of cancer (20 genes), p38 MAPK signaling (10 genes), glucocorticoid receptor signaling (18 genes), and docosahexaenoic acid (DHA) signaling (6 genes), with a q-value < 0.05 **(**Table 3 and Additional file 3: Table S3). One of them, the DHA signaling pathway (Fig. 4), has been reported to be associated with DLB pathology; high levels of alpha-synuclein oligomers were induced by high levels of DHA in vitro and in vivo [24]. This result suggests that six genes (PNPLA2, PIK3C2B, PIK3R2, GSK3A, GSK3B, and BCL2L1) included in this DHA signaling pathway, could be associated with DLB pathology (Fig. 4).

**Table 3:** Canonical pathways associated with DLB pathology

Canonical pathway	#genes related	q-value
protein kinase a signaling	21	7.08E-3
ERK/MAPK signaling	14	7.08E-3
molecular mechanisms of cancer	20	7.6E-3
p38 MAPK signaling	10	7.6E-3
glucocorticoid receptor signaling	18	8.92E-3
docosahexaenoic acid (DHA) signaling	6	3.19E-2

### Gene-based association studies using large numbers of SNP markers

To check the genetic associations of the six genes in the DHA signaling pathway, we examined genetic differences using single nucleotide polymorphism (SNP) markers from 69 DLB cases and 2008 controls. A gene-based association test of rare variants, SNP-set Kernel Association Test (SKAT) [25], was applied to the gene coding sequence six genes including 1 Mb of sequence up and downstream, since expression quantitative trait loci (eQTL) SNPs [26] have a major effect on gene expression regulation. Several thousand SNPs were used for the association tests, which showed statistically significant association with BCL2L1 (*p*-value = 0.012) and PIK3R2 (p-value = 0.021) (Table 4). Furthermore, the expression of these two genes was observed in several brain tissues in the Genotype-Tissue Expression (GTEx) database [27].

**Table 4:** Gene-based association studies for the six genes in the DHA signaling pathway

Gene on pathway	Gene symbol	#SNPs	*p*-value
PNPLA	PNPLA2	376	0.059
PI3K	PIK3C2B	560	0.915
	PIK3R2	364	0.021*
GSK3	GSK3A	174	0.371
	GSK3B	429	0.451
BCL-XL	BCL2L1	135	0.012*

*statistically significant association

## Discussion

Early diagnosis and therapeutic intervention could prevent severe disease manifestations in patients suffering from several diseases including DLB, and miRNAs have attracted a lot of attention as novel biomarkers [28–30]. In fact, risk prediction models using miRNA biomarkers have been developed for early diagnosis prediction in several types of dementia [9], including sporadic AD [31], as well as cancers [32, 33]. However, more accurate prediction models are required for practical clinical use.

To construct a more accurate risk prediction model for DLB, we, in this study, used comprehensive miRNA expression data of serum samples and applied several ML methods. We investigated which combination of ML method and miRNA sets resulted in the best predictive model. We found that the GBDT method achieved the highest accuracy among four ML methods examined, although the performance of all ML methods was similar (Fig. 2). Other studies have recently reported the powerful classification performance of GBDT [34], which produces a prediction model in the form of an ensemble of weak prediction models (decision trees), constructs the model in a stage-wise fashion, and generalizes them by allowing optimization of an arbitrary differentiable loss function. It has also been reported that for this ML model, the arbitrary customization of the loss function contributes to the recent success of prediction models [35].

Our final GBDT risk prediction model was constructed using 216 pre-selected top-ranked miRNAs, selected through a logistic regression method, and three clinical features. However, only 180 of the 216 miRNAs contributed to the risk prediction model construction as efficient features. Of the 180 miRNAs, 7 showed a high feature importance in the final GBDT model. GSEA using the target gens of the 7 miRNAs detected a significantly enriched biological pathway, the DHA signaling pathway, which has been previously reported to be associated with DLB pathology [24]. In particular, six target genes were involved in the biological pathway, two of which, BCL2L1 and PIK3R2, were further supported by gene-based association studies using a large number of SNP markers. BCL2L1 belongs to the family of BDL-2 proteins, which is involved in not only in the control of apoptosis, but also in mitochondrial damage protection [36], modulation of immune response [37], and DNA repair [38]. Borras et al. have reported that the over-expression of BCL2L1 in PBMCs was confirmed in centenarians, compared with septuagenarians and young people [39]. This evidence supports that BCL2L1 plays an important role in healthy aging. In other words, defects in BCL2L1 could exert an adverse influence on the healthy aging (e.g. cognitive impairment). On the other hand, PIK3R2 (phosphoinositide-3-kinase regulatory subunit 2) is a lipid kinase that functions in growth signaling pathways and a known as a tumor suppressor gene [40]. There are no reports of it being associated with DLB pathology. However, Shu et al. reported that the PI3K/ANK pathway containing PIK3R2 is involved in cognitive impairment [41]. These miRNAs were also used to perform DIANA-miRPath v3.0 [42], a web-based functional analysis tool incorporating biological pathways, and three statistically significant Kyoto Encyclopedia of Genes and Genomes (KEGG) biological pathways [43, 44] were detected with a q-value < 0.001: Metabolism of xenobiotics by cytochrome P450 (8 genes), Vasopressin-regulated water reabsorption (14 genes), and thyroid hormone signaling pathway (25 genes) (Additional file 4: Table S4). One of them, the thyroid hormone signaling pathway, has been reported to be associated with neurodegenerative diseases; the administration of thyroid hormone in AD model mice prevented cognitive deficit and improved the neurological function [45]. In future work, we will perform further refinement of our model, and investigations with larger sample size will further validate the effectiveness of this classifier.

The most efficient features have a weaker correlation between the ranking of feature importance of the final method and the top-ranked miRNAs than we expected (Spearman’s ρ =0.21). This implies that there is still room for improvement in our prediction model. One way may be to integrate functional units, such as metabolic pathways, into our final risk prediction model, as miRNAs with high feature importance in the GBDT were associated with several biological pathways. Another way may be to integrate interactions among miRNAs into our final risk prediction model. Interaction effects have been reported to increase the power of risk prediction models [46]. Finally, artificial intelligence (AI) technology, in particular deep learning, is a recent and fast-growing field of machine learning. AI technology could also contribute to an improvement of this risk prediction model. Some potential applications have been proposed for novel diagnostic and treatment options in medical imaging and genomics [47, 48]. However, this technology relies on large amounts of data to learn automatically, and it has certain advantages for dealing with big data [49, 50]. At least several thousand unique training data sets would be required for successful application [50]. Although at present, our data sets were too small to effectively apply these AI technologies, we expect that these technologies will contribute to improvement of our prediction model in the future.

Next generation sequencing technology (NGS) has enabled comprehensive detection of coding and non-coding RNAs as well as genetic variants. Integrative analysis of these genetic variations and gene expressions, such as expression quantitative trait loci (eQTL), has revealed potential target genes for associations of genetic susceptibility risk loci. We believe that these omics data would also play an important role for improvement of risk prediction models.

## Conclusions

In this study, we investigated potential miRNA biomarkers using serum miRNA expression and constructed an optimal risk prediction model using several machine learning methods. The final risk prediction model based on a GBDT achieved an accuracy of 0.829 on an independent test set. GSEA of the miRNA candidate target genes revealed 6 functional genes in the DHA signaling pathway associated with DLB pathology. For two of them (BCL2L1 and PIK3R2), this was further supported by gene-based association studies using a large numbers of SNP markers. Our study provides an effective tool for DLB classification, and with further improvement, such as integrative analyses of genomic and/or transcriptomic data, it has the potential to contribute to practical clinical application in DLB.

## Supplementary information

**Additional file 1: Table S1.** Hyperparameter values in the final GBDT model when using *μ*HEM algorithm.

**Additional file 2: Table S2.** All features used in the final GBDT risk prediction model.

**Additional file 3: Table S3.** Genes and the annoatation related to the canonical pathways.

**Additional file 4: Table S4.** Genes including in the KEGG pathways.

## Data Availability

All microarray data used in this study are publicly available through the GEO database accession number GSE120584 (https://www.ncbi.nlm.nih.gov/geo/). Other datasets generated in this study are provided from the corresponding author on reasonable request.

## Abbreviations

AD: Alzheimer’s disease
AI: Artificial intelligence
ChEIs: Cholinesterase inhibitors
DHA: Docosahexaenoic acid
DLB: Dementia with Lewy bodies
eQTL: Expression quantitative trait loci
FN: False negatives
FP: False positives
GBDT: Gradient boosted decision tree
GEO: Gene Expression Omnibus
GSEA: Gene set enrichment analysis
GTEx: Genotype-Tissue Expression
LBD: Lewy body dementia
MAF: Minor allele frequency
miRDB: microRNA Target Prediction and Functional Study Database
miRNAs: MicroRNAs
ML: Machine learning
MMSE: Mini-Mental State Examination
NC: Normal controls
NGS: Next generation sequencing technology
PCA: Principal component analysis
PDD: Parkinson’s disease dementia
RF: Random forest
SKAT: SNP-set Kernel Association Test
SNP: Single nucleotide polymorphism
SVM: Support vector machine
TN: True negatives
TP: True positives
α Syn: α-synuclein
