# Supplementary material for: A comparison of machine learning classifiers for dementia with Lewy bodies using miRNA expression data
Source: BMC Med Genomics. 2019 Oct 30;12:150. doi: 10.1186/s12920-019-0607-3 (PMC6822471; doi:10.1186/s12920-019-0607-3)
Supplement: Supplementary file 1 — Additional file 1: Table S1. Hyperparameter values in the final GBDT model when using μHEM algorithm. [file 12920_2019_607_MOESM1_ESM.pdf]

Supplementary Table S1. Hyperparameter values in the final GBDT model when using μHEM algorithm

#top-ranked miRNA	Hyperparameter	Value
330	max_{depth}	3
	n_{tree}	700
	min_{split}	15
	min_{sample}	1
	learning rate	0.01
