# Supplementary material for: A comparison of machine learning classifiers for dementia with Lewy bodies using miRNA expression data
Source: BMC Med Genomics. 2019 Oct 30;12:150. doi: 10.1186/s12920-019-0607-3 (PMC6822471; doi:10.1186/s12920-019-0607-3)
Supplement: Supplementary file 2 — Additional file 2: Table S2. All features used in the final GBDT risk prediction model. [file 12920_2019_607_MOESM2_ESM.pdf]

Supplementary Table S2. All features used in the final GBDT risk prediction model

feature	importance	rank	hsaID	#target gene
Age	0.087894778	-	-	0
MIMAT0014984	0.021824435	9	hsa-miR-3122	13
MIMAT0027624	0.019877076	8	hsa-miR-6861-3p	9
MIMAT0016852	0.016797957	75	hsa-miR-4298	21
MIMAT0023713	0.016200595	18	hsa-miR-6088	45
MIMAT0019849	0.015702967	3	hsa-miR-4728-5p	278
MIMAT0022491	0.015538316	22	hsa-miR-5698	82
MIMAT0007882	0.015001331	141	hsa-miR-1909-5p	6
MIMAT0007400	0.013176565	151	hsa-miR-1538	0
MIMAT0027551	0.013062134	6	hsa-miR-6825-3p	1
MIMAT0016875	0.012469331	5	hsa-miR-4323	26
MIMAT0027559	0.012363385	35	hsa-miR-6829-3p	22
MIMAT0027468	0.012205476	210	hsa-miR-6784-5p	1
MIMAT0027455	0.011532623	4	hsa-miR-6777-3p	9
MIMAT0019893	0.01082506	12	hsa-miR-371b-3p	9
MIMAT0022731	0.010735721	1	hsa-miR-3184-3p	17
MIMAT0007884	0.010672442	97	hsa-miR-1910-5p	27
MIMAT0011775	0.009909107	164	hsa-miR-2276-3p	31
MIMAT0006765	0.009562143	88	hsa-miR-1825	28
MIMAT0005865	0.009191851	98	hsa-miR-1202	23
MIMAT0027453	0.009084551	180	hsa-miR-6776-3p	16
MIMAT0027512	0.009081382	86	hsa-miR-6806-5p	21
MIMAT0014995	0.009000495	26	hsa-miR-3130-5p	11
MIMAT0018099	0.008788992	100	hsa-miR-3675-3p	22
MIMAT0022966	0.008498452	124	hsa-miR-3617-3p	39
MIMAT0027549	0.008259965	118	hsa-miR-6824-3p	19
MIMAT0026718	0.008112199	127	hsa-miR-874-5p	30
MIMAT0027593	0.007973836	76	hsa-miR-6846-3p	8
MIMAT0010133	0.00794066	138	hsa-miR-2110	61
MIMAT0015024	0.007660483	49	hsa-miR-3151-5p	41
MIMAT0027465	0.006885697	108	hsa-miR-6782-3p	10
MIMAT0027503	0.006878968	21	hsa-miR-6801-3p	21
MIMAT0019978	0.00674175	125	hsa-miR-4800-5p	4
MIMAT0027542	0.006648343	216	hsa-miR-6821-5p	0
MIMAT0015054	0.006126751	206	hsa-miR-3177-3p	2
MIMAT0009203	0.006094551	134	hsa-miR-449b-3p	54
MIMAT0027641	0.005720572	27	hsa-miR-6870-3p	10
MIMAT0018065	0.005422034	61	hsa-miR-3646	369
MIMAT0027689	0.005397098	130	hsa-miR-6894-3p	21
MIMAT0027635	0.005317276	16	hsa-miR-6867-3p	30
MIMAT0003283	0.00526068	32	hsa-miR-615-3p	1

MIMAT0028220	0.005215414	87	hsa-miR-7155-5p	21
MIMAT0027495	0.00520274	31	hsa-miR-6797-3p	9
MIMAT0004970	0.005135993	56	hsa-miR-920	14
MIMAT0031000	0.005064258	107	hsa-miR-8073	39
MIMAT0016915	0.004559906	33	hsa-miR-4284	28
MIMAT0022698	0.004492144	70	hsa-miR-345-3p	74
MIMAT0013518	0.004477241	19	hsa-miR-2682-3p	18
MIMAT0000773	0.004380726	175	hsa-miR-346	10
MIMAT0016923	0.004175919	165	hsa-miR-4329	22
MIMAT0004979	0.004139046	69	hsa-miR-936	37
MIMAT0027386	0.003851197	163	hsa-miR-6742-3p	17
MIMAT0027688	0.003744408	129	hsa-miR-6894-5p	3
MIMAT0027576	0.003719751	119	hsa-miR-6837-5p	73
MIMAT0019216	0.003593537	186	hsa-miR-3187-5p	67
MIMAT0019738	0.003538177	114	hsa-miR-4664-3p	0
MIMAT0012735	0.003440228	99	hsa-miR-718	1
MIMAT0002853	0.003327108	171	hsa-miR-519d-3p	196
MIMAT0019708	0.003270484	145	hsa-miR-4646-3p	14
MIMAT0016886	0.003210159	204	hsa-miR-4252	42
MIMAT0021124	0.002996596	155	hsa-miR-5193	31
MIMAT0000447	0.002792641	214	hsa-miR-134-5p	16
MIMAT0003888	0.002749001	83	hsa-miR-766-3p	28
MIMAT0003307	0.002700439	92	hsa-miR-637	54
MIMAT0005593	0.002629835	101	hsa-miR-1238-3p	0
MIMAT0028214	0.002578447	80	hsa-miR-7152-5p	41
MIMAT0011778	0.002552951	93	hsa-miR-2278	60
MIMAT0027615	0.002552385	179	hsa-miR-6857-3p	13
MIMAT0027591	0.002540493	11	hsa-miR-6845-3p	29
MIMAT0028120	0.002458635	66	hsa-miR-7111-3p	29
MIMAT0018200	0.002458024	211	hsa-miR-3925-5p	49
MIMAT0000228	0.002449679	77	hsa-miR-198	22
MIMAT0000770	0.002432711	190	hsa-miR-133b	65
APOE	0.002371952	-	-	0
MIMAT0019837	0.002336457	47	hsa-miR-4722-3p	37
MIMAT0027509	0.002335804	106	hsa-miR-6804-3p	18
MIMAT0027403	0.00230331	182	hsa-miR-6751-3p	28
MIMAT0018360	0.00223285	146	hsa-miR-3944-3p	0
MIMAT0028118	0.002213208	158	hsa-miR-7110-3p	25
MIMAT0006790	0.002174134	23	hsa-miR-675-3p	13
MIMAT0019798	0.002151626	139	hsa-miR-4701-5p	26
MIMAT0027675	0.002128047	82	hsa-miR-6887-3p	85
MIMAT0019792	0.002128041	2	hsa-miR-4697-3p	25
MIMAT0028126	0.002080059	53	hsa-miR-7114-3p	9

MIMAT0019211	0.002071174	94	hsa-miR-3158-5p	58
MIMAT0027460	0.001990138	173	hsa-miR-6780a-5p	79
MIMAT0007888	0.001970147	112	hsa-miR-1913	39
MIMAT0026474	0.001961809	117	hsa-miR-208a-5p	76
MIMAT0027384	0.001950355	65	hsa-miR-6741-3p	1
MIMAT0027573	0.001947175	126	hsa-miR-6780b-3p	10
MIMAT0027416	0.001913718	198	hsa-miR-6758-5p	52
MIMAT0015057	0.001883122	142	hsa-miR-3180-5p	73
MIMAT0004693	0.001813244	178	hsa-miR-330-5p	52
MIMAT0021043	0.001812507	143	hsa-miR-5010-5p	5
MIMAT0022939	0.001791829	78	hsa-miR-939-3p	65
MIMAT0027421	0.001728277	121	hsa-miR-6760-3p	23
MIMAT0028116	0.001653546	60	hsa-miR-7109-3p	18
MIMAT0027389	0.0015751	41	hsa-miR-6744-5p	7
MIMAT0027405	0.001570694	150	hsa-miR-6752-3p	52
MIMAT0027651	0.001523438	29	hsa-miR-6875-3p	81
MIMAT0027655	0.001504628	58	hsa-miR-6877-3p	19
MIMAT0027662	0.001494797	196	hsa-miR-6881-5p	11
MIMAT0016906	0.001490293	174	hsa-miR-4274	21
MIMAT0027505	0.001474516	199	hsa-miR-6802-3p	11
MIMAT0027661	0.001449805	68	hsa-miR-6880-3p	0
MIMAT0021127	0.001443025	85	hsa-miR-5195-3p	77
MIMAT0028221	0.001424907	177	hsa-miR-7155-3p	8
MIMAT0030413	0.001376039	215	hsa-miR-4433b-5p	35
MIMAT0005592	0.001292809	192	hsa-miR-1237-3p	18
MIMAT0019854	0.00127643	67	hsa-miR-4731-3p	54
MIMAT0018958	0.001242387	208	hsa-miR-4440	0
MIMAT0025479	0.001225613	213	hsa-miR-6511a-3p	14
MIMAT0000227	0.001206704	137	hsa-miR-197-3p	31
MIMAT0018350	0.001196625	116	hsa-miR-3935	10
MIMAT0027088	0.001166921	195	hsa-miR-5189-3p	15
MIMAT0019826	0.001151499	7	hsa-miR-4716-5p	20
MIMAT0027479	0.001118468	46	hsa-miR-6789-3p	1
MIMAT0027489	0.001108678	185	hsa-miR-6794-3p	37
MIMAT0027567	0.001092919	193	hsa-miR-6833-3p	84
MIMAT0022499	0.001061285	176	hsa-miR-5705	0
MIMAT0027519	0.001060949	166	hsa-miR-6809-3p	147
MIMAT0018001	0.001047539	140	hsa-miR-3620-3p	15
MIMAT0016888	0.001044908	37	hsa-miR-4326	10
MIMAT0017992	0.001031025	115	hsa-miR-3614-5p	19
MIMAT0005591	0.001014148	120	hsa-miR-1236-3p	52
MIMAT0004671	0.001008845	133	hsa-miR-194-3p	32
MIMAT0025845	0.001005543	24	hsa-miR-6716-3p	1

MIMAT0004780	0.00099124	14	hsa-miR-532-3p	18
MIMAT0016921	0.000984199	63	hsa-miR-4290	20
MIMAT0022726	0.00098013	30	hsa-miR-1306-5p	13
MIMAT0005588	0.000971383	13	hsa-miR-1233-3p	21
MIMAT0016909	0.000961014	96	hsa-miR-4279	130
MIMAT0027419	0.000924299	162	hsa-miR-6759-3p	15
MIMAT0027370	0.000918057	136	hsa-miR-6734-3p	48
MIMAT0027439	0.000901033	15	hsa-miR-6769a-3p	14
MIMAT0027547	0.000862269	157	hsa-miR-6823-3p	12
MIMAT0027492	0.000833376	189	hsa-miR-6796-5p	33
MIMAT0027530	0.000818346	187	hsa-miR-6815-5p	22
MIMAT0004950	0.000817037	36	hsa-miR-877-3p	36
MIMAT0022496	0.000788065	144	hsa-miR-5703	60
MIMAT0007349	0.000778128	95	hsa-miR-1471	0
MIMAT0003881	0.000761576	20	hsa-miR-668-3p	5
MIMAT0027026	0.000756321	89	hsa-miR-3151-3p	9
MIMAT0005584	0.000692922	48	hsa-miR-1229-3p	14
MIMAT0027469	0.000687313	91	hsa-miR-6784-3p	14
MIMAT0027366	0.000684974	160	hsa-miR-6732-3p	25
MIMAT0009451	0.000676382	38	hsa-miR-1976	20
MIMAT0019940	0.000673698	73	hsa-miR-4436b-5p	37
MIMAT0027103	0.000663485	207	hsa-miR-5699-5p	24
MIMAT0027027	0.00065736	84	hsa-miR-3192-3p	15
MIMAT0018359	0.000650419	103	hsa-miR-3943	18
MIMAT0028110	0.000600206	17	hsa-miR-7106-3p	15
MIMAT0027569	0.000569534	169	hsa-miR-6834-3p	11
MIMAT0028230	0.000566278	45	hsa-miR-7160-5p	59
MIMAT0027475	0.000548472	55	hsa-miR-6787-3p	29
MIMAT0027679	0.000547346	39	hsa-miR-6889-3p	21
MIMAT0003312	0.000539481	154	hsa-miR-642a-5p	36
MIMAT0019883	0.000533131	72	hsa-miR-4747-3p	0
MIMAT0019209	0.00052766	54	hsa-miR-3156-3p	25
MIMAT0005459	0.000506625	149	hsa-miR-1224-3p	25
MIMAT0027425	0.000499545	201	hsa-miR-6762-3p	39
MIMAT0002176	0.000477381	113	hsa-miR-485-3p	70
MIMAT0027681	0.000439331	181	hsa-miR-6890-3p	22
MIMAT0027354	0.000400628	52	hsa-miR-6726-3p	0
MIMAT0027457	0.00038657	197	hsa-miR-6778-3p	75
MIMAT0027423	0.00034053	109	hsa-miR-6761-3p	0
MIMAT0000231	0.000331266	153	hsa-miR-199a-5p	52
MIMAT0027631	0.000327134	105	hsa-miR-6865-3p	9
MIMAT0027473	0.000315883	102	hsa-miR-6786-3p	1
MIMAT0027603	0.000293512	159	hsa-miR-6851-3p	7

MIMAT0027374	0.000284492	168	hsa-miR-6736-3p	42
MIMAT0027630	0.000268711	147	hsa-miR-6865-5p	25
MIMAT0015052	0.000259103	74	hsa-miR-3175	83
MIMAT0027671	0.000243487	28	hsa-miR-6885-3p	146
MIMAT0018004	0.000220391	51	hsa-miR-3622a-3p	30
MIMAT0022736	0.000218747	122	hsa-miR-642b-5p	20
MIMAT0004677	0.000189608	184	hsa-miR-34c-3p	18
MIMAT0010367	0.000174638	194	hsa-miR-764	13
MIMAT0027415	0.000168969	152	hsa-miR-6757-3p	40
MIMAT0016896	0.000168048	34	hsa-miR-4268	12
MIMAT0027401	9.40E-05	111	hsa-miR-6750-3p	20
MIMAT0026740	3.49E-05	167	hsa-miR-1250-3p	111
Sex	0	-	-	0
MIMAT0031012	0	188	hsa-miR-8085	110
MIMAT0027687	0	25	hsa-miR-6893-3p	40
MIMAT0027626	0	62	hsa-miR-6862-3p	15
MIMAT0027619	0	57	hsa-miR-6859-3p	5
MIMAT0027617	0	10	hsa-miR-6858-3p	18
MIMAT0027597	0	200	hsa-miR-6848-3p	19
MIMAT0027570	0	132	hsa-miR-6835-5p	6
MIMAT0027543	0	183	hsa-miR-6821-3p	4
MIMAT0027527	0	43	hsa-miR-6813-3p	7
MIMAT0027507	0	59	hsa-miR-6803-3p	0
MIMAT0027501	0	110	hsa-miR-6800-3p	13
MIMAT0027485	0	161	hsa-miR-6792-3p	42
MIMAT0027459	0	64	hsa-miR-6779-3p	34
MIMAT0027409	0	156	hsa-miR-6754-3p	10
MIMAT0027395	0	128	hsa-miR-6747-3p	29
MIMAT0026475	0	135	hsa-miR-210-5p	17
MIMAT0025848	0	40	hsa-miR-6511b-3p	14
MIMAT0022297	0	104	hsa-miR-5589-5p	18
MIMAT0021129	0	44	hsa-miR-5196-3p	10
MIMAT0021024	0	131	hsa-miR-5002-3p	9
MIMAT0019850	0	90	hsa-miR-4728-3p	40
MIMAT0019843	0	172	hsa-miR-4725-5p	17
MIMAT0019839	0	79	hsa-miR-4723-3p	16
MIMAT0019789	0	42	hsa-miR-4695-3p	10
MIMAT0019707	0	170	hsa-miR-4646-5p	54
MIMAT0019702	0	123	hsa-miR-4642	21
MIMAT0018005	0	71	hsa-miR-3622b-5p	15
MIMAT0017982	0	81	hsa-miR-3605-3p	0
MIMAT0005872	0	212	hsa-miR-1207-3p	40
MIMAT0004687	0	50	hsa-miR-371a-5p	23

MIMAT0004611	0	202	hsa-miR-185-3p	88
MIMAT0003233	0	209	hsa-miR-551b-3p	3
MIMAT0003214	0	148	hsa-miR-551a	3
MIMAT0002173	0	203	hsa-miR-483-3p	12
MIMAT0000690	0	205	hsa-miR-296-5p	27
MIMAT0000450	0	191	hsa-miR-149-5p	44
