# Supplementary material for: A comparison of machine learning classifiers for dementia with Lewy bodies using miRNA expression data
Source: BMC Med Genomics. 2019 Oct 30;12:150. doi: 10.1186/s12920-019-0607-3 (PMC6822471; doi:10.1186/s12920-019-0607-3)
Supplement: Supplementary file 3 — Additional file 3: Table S3. Genes and the annoatation related to the canonical pathways. [file 12920_2019_607_MOESM3_ESM.pdf]

Supplementary Table S3. Genes and the annoatation related to the canonical pathways

Canonical pathway	Symbol	Entrez Gene Name	Entrez Gene ID	Location
protein kinase a signaling	ADD3	adducin 3	120	Cytoplasm
	AKAP6	A-kinase anchoring protein 6	9472	Nucleus
	AKAP13	A-kinase anchoring protein 13	11214	Cytoplasm
	CREB1	cAMP responsive element binding protein 1	1385	Nucleus
	DUSP2	dual specificity phosphatase 2	1844	Nucleus
	DUSP4	dual specificity phosphatase 4	1846	Nucleus
	DUSP8	dual specificity phosphatase 8	1850	Nucleus
	ELK1	ELK1, ETS transcription factor	2002	Nucleus
	EYA3	EYA transcriptional coactivator and phosphatase 3	2140	Nucleus
	GSK3A	glycogen synthase kinase 3 alpha	2931	Nucleus
	GSK3B	glycogen synthase kinase 3 beta	2932	Nucleus
	NFAT5	nuclear factor of activated T cells 5	10725	Nucleus
	NGFR	nerve growth factor receptor	4804	Plasma Membrane
	PDE4A	phosphodiesterase 4A	5141	Cytoplasm
	PRKACA	protein kinase cAMP-activated catalytic subunit alpha	5566	Cytoplasm
	PTPN3	protein tyrosine phosphatase, non-receptor type 3	5774	Cytoplasm
	PTPRA	protein tyrosine phosphatase, receptor type A	5786	Plasma Membrane
	PTPRJ	protein tyrosine phosphatase, receptor type J	5795	Plasma Membrane
	SMAD3	SMAD family member 3	4088	Nucleus
	YWHAQ	tyrosine 3-monooxygenasetryptophan 5-monooxygenase activation protein theta	10971	Cytoplasm
	YWHAZ	tyrosine 3-monooxygenasetryptophan 5-monooxygenase activation protein zeta	7534	Cytoplasm
ERK/MAPK signaling	CREB1	cAMP responsive element binding protein 1	1385	Nucleus
	DUSP2	dual specificity phosphatase 2	1844	Nucleus
	DUSP4	dual specificity phosphatase 4	1846	Nucleus
	ELK1	ELK1, ETS transcription factor	2002	Nucleus
	HSPB7	heat shock protein family B (small) member 7	27129	Cytoplasm
	ITGA3	integrin subunit alpha 3	3675	Plasma Membrane
	MKNK2	MAP kinase interacting serinethreonine kinase 2	2872	Cytoplasm
	PIK3C2B	phosphatidylinositol-4-phosphate 3-kinase catalytic subunit type 2 beta	5287	Cytoplasm
	PIK3R2	phosphoinositide-3-kinase regulatory subunit 2	5296	Cytoplasm
	PLA2G4E	phospholipase A2 group IVE	123745	Cytoplasm
	PRKACA	protein kinase cAMP-activated catalytic subunit alpha	5566	Cytoplasm
	SRF	serum response factor	6722	Nucleus
	YWHAQ	tyrosine 3-monooxygenasetryptophan 5-monooxygenase activation protein theta	10971	Cytoplasm
	YWHAZ	tyrosine 3-monooxygenasetryptophan 5-monooxygenase activation protein zeta	7534	Cytoplasm
molecular mechanisms of cancer	BCL2L1	BCL2 like 1	598	Cytoplasm
	CDK14	cyclin dependent kinase 14	5218	Nucleus
	CDKN1A	cyclin dependent kinase inhibitor 1A	1026	Nucleus
	ELK1	ELK1, ETS transcription factor	2002	Nucleus
	FZD7	frizzled class receptor 7	8324	Plasma Membrane
	GNAT1	G protein subunit alpha transducin 1	2779	Plasma Membrane
	GSK3A	glycogen synthase kinase 3 alpha	2931	Nucleus
	GSK3B	glycogen synthase kinase 3 beta	2932	Nucleus
	ITGA3	integrin subunit alpha 3	3675	Plasma Membrane
	LRP1	LDL receptor related protein 1	4035	Plasma Membrane
	PA2G4	proliferation-associated 2G4	5036	Nucleus
	PIK3C2B	phosphatidylinositol-4-phosphate 3-kinase catalytic subunit type 2 beta	5287	Cytoplasm
	PIK3R2	phosphoinositide-3-kinase regulatory subunit 2	5296	Cytoplasm
	PRKACA	protein kinase cAMP-activated catalytic subunit alpha	5566	Cytoplasm
	RALBP1	ralA binding protein 1	10928	Cytoplasm
	RHOB	ras homolog family member B	388	Cytoplasm
	SMAD3	SMAD family member 3	4088	Nucleus
	SUFU	SUFU negative regulator of hedgehog signaling	51684	Nucleus
	SYNGAP1	synaptic Ras GTPase activating protein 1	8831	Plasma Membrane
	WNT1	Wnt family member 1	7471	Extracellular Space
p38 MAPK signaling	CREB1	cAMP responsive element binding protein 1	1385	Nucleus
	ELK1	ELK1, ETS transcription factor	2002	Nucleus
	HSPB7	heat shock protein family B (small) member 7	27129	Cytoplasm
	MAPT	microtubule associated protein tau	4137	Plasma Membrane
	MEF2C	myocyte enhancer factor 2C	4208	Nucleus
	MEF2D	myocyte enhancer factor 2D	4209	Nucleus
	MKNK2	MAP kinase interacting serinethreonine kinase 2	2872	Cytoplasm
	PLA2G4E	phospholipase A2 group IVE	123745	Cytoplasm
	RPS6KA2	ribosomal protein S6 kinase A2	6196	Nucleus
	SRF	serum response factor	6722	Nucleus
glucocorticoid receptor signaling	AR	androgen receptor	367	Nucleus
	BCL2L1	BCL2 like 1	598	Cytoplasm
	CDKN1A	cyclin dependent kinase inhibitor 1A	1026	Nucleus
	CREB1	cAMP responsive element binding protein 1	1385	Nucleus
	ELK1	ELK1, ETS transcription factor	2002	Nucleus
	KRT75	keratin 75	9119	Cytoplasm
	KRT222	keratin 222	125113	Other
	NCOA1	nuclear receptor coactivator 1	8648	Nucleus
	NFAT5	nuclear factor of activated T cells 5	10725	Nucleus
	PIK3C2B	phosphatidylinositol-4-phosphate 3-kinase catalytic subunit type 2 beta	5287	Cytoplasm
	PIK3R2	phosphoinositide-3-kinase regulatory subunit 2	5296	Cytoplasm
	POLR2E	RNA polymerase II subunit E	5434	Nucleus
	POU2F2	POU class 2 homeobox 2	5452	Nucleus
	PRKACA	protein kinase cAMP-activated catalytic subunit alpha	5566	Cytoplasm
	SMAD3	SMAD family member 3	4088	Nucleus
	SMARCC2	SWISNF related, matrix associated, actin dependent regulator of chromatin subfamily c member 2	6601	Nucleus
	SMARCD1	SWISNF related, matrix associated, actin dependent regulator of chromatin, subfamily d, member 1	6602	Nucleus
	TAF5	TATA-box binding protein associated factor 5	6877	Nucleus
docosahexaenoic acid (DHA) signaling	BCL2L1	BCL2 like 1	598	Cytoplasm
	GSK3A	glycogen synthase kinase 3 alpha	2931	Nucleus
	GSK3B	glycogen synthase kinase 3 beta	2932	Nucleus
	PIK3C2B	phosphatidylinositol-4-phosphate 3-kinase catalytic subunit type 2 beta	5287	Cytoplasm
	PIK3R2	phosphoinositide-3-kinase regulatory subunit 2	5296	Cytoplasm
	PNPLA2	patatin like phospholipase domain containing 2	57104	Cytoplasm
