# Supplementary material for: A comparison of machine learning classifiers for dementia with Lewy bodies using miRNA expression data
Source: BMC Med Genomics. 2019 Oct 30;12:150. doi: 10.1186/s12920-019-0607-3 (PMC6822471; doi:10.1186/s12920-019-0607-3)
Supplement: Supplementary file 4 — Additional file 4: Table S4. Genes including in the KEGG pathways. [file 12920_2019_607_MOESM4_ESM.pdf]

Supplementary Table S4. Genes including in the KEGG pathways

KEGG pathway	Symbol
Metabolism of xenobiotics by cytochrome P450	CYP1A2
	GSTM4
	GSTO2
	GSTM1
	GSTM3
	ALDH3B2
	GSTM2
	GSTA4
Vasopressin-regulated water reabsorption	GNAS
	CREB5
	DCTN2
	CREB1
	PRKACA
	ARHGDIA
	PRKX
	DYNLL2
	CREB3L2
	CREB3L1
	RAB11B
	RAB5B
	AQP2
	VAMP2
Thyroid hormone signaling pathway	ACTB
	GSK3B
	PRKCA
	NRAS
	WNT4
	THRA
	PLCZ1
	KRAS
	TP53
	MED1
	MED16
	SLC9A1
	PIK3R3
	PRKACA
	ATP1A4
	PIK3CG
	PRKX
	CASP9
	THRB
	SLC16A2
	SLC2A1

	MDM2
	NCOA1
	ITGB3
	PIK3R2
